# Determinants of quality of shared sanitation facilities in informal settlements: case study of Kisumu, Kenya

**DOI:** 10.1186/s12889-016-4009-6

**Published:** 2017-01-11

**Authors:** Sheillah Simiyu, Mark Swilling, Sandy Cairncross, Richard Rheingans

**Affiliations:** 1 School of Public Leadership, Stellenbosch University, Private Bag, X1, Matieland, 7602 Stellenbosch South Africa; 2 Department of Disease Control, London School of Hygiene and Tropical Medicine, Keppel Street, London, WC1E 7HT UK; 3 Department of Environmental and Global Health, University of Florida, Gainesville, FL 32611 USA; 4 Great Lakes University of Kisumu (GLUK), Box 2224-40100, Kisumu, Kenya; 5 Sustainable Development Department, Appalachian State University, 287 Rivers St, Boone, NC USA

**Keywords:** Sanitation quality, Common pool resources, Management principles, Collective action, Behaviour

## Abstract

**Background:**

Shared facilities are not recognised as improved sanitation due to challenges of maintenance as they easily can be avenues for the spread of diseases. Thus there is need to evaluate the quality of shared facilities, especially in informal settlements, where they are commonly used. A shared facility can be equated to a common good whose management depends on the users. If users do not work collectively towards keeping the facility clean, it is likely that the quality may depreciate due to lack of maintenance. This study examined the quality of shared sanitation facilities and used the common pool resource (CPR) management principles to examine the determinants of shared sanitation quality in the informal settlements of Kisumu, Kenya.

**Methods:**

Using a multiple case study design, the study employed both quantitative and qualitative methods. In both phases, users of shared sanitation facilities were interviewed, while shared sanitation facilities were inspected. Shared sanitation quality was a score which was the dependent variable in a regression analysis. Interviews during the qualitative stage were aimed at understanding management practices of shared sanitation users. Qualitative data was analysed thematically by following the CPR principles.

**Results:**

Shared facilities, most of which were dirty, were shared by an average of eight households, and their quality decreased with an increase in the number of households sharing. The effect of numbers on quality is explained by behaviour reflected in the CPR principles, as it was easier to define boundaries of shared facilities when there were fewer users who cooperated towards improving their shared sanitation facility. Other factors, such as defined management systems, cooperation, collective decision making, and social norms, also played a role in influencing the behaviour of users towards keeping shared facilities clean and functional.

**Conclusion:**

Apart from hardware factors, quality of shared sanitation is largely due to group behaviour of users. The CPR principles form a crucial lens through which the dynamics of shared sanitation facilities in informal settlements can be understood. Development and policy efforts should incorporate group behaviour as they determine the quality of shared sanitation facilities.

## Background

The sanitation target of the sixth Sustainable Development Goal(SDG) is to achieve access to adequate and equitable sanitation for all by 2030. Due to increasing urbanisation and informality, however, providing adequate sanitation in informal settlements is increasingly becoming a challenge [1]. Inadequate household sanitation facilities in informal settlements force residents to share the few available facilities, a practice that some authors have proposed as being the most practical alternative [2, 3]. In the classification of sanitation facilities, however, the Joint Monitoring Program (JMP) of the World Health Organization (WHO) and the United Nations Children’s Fund (UNICEF) does not classify shared sanitation facilities as ‘improved’ facilities due to concerns related to, among others, cleanliness and maintenance [4].

In addition to cleanliness and maintenance, studies have also highlighted the importance of aspects such as hygienic status of sanitation facilities, state of the superstructure, presence of smell, presence of flies, and the state of the slab (especially in the case of pit latrines) in defining the quality of shared sanitation [5–10]. What is evident from these studies is that quality of sanitation facilities is determined by maintenance practices such as cleaning or lack thereof.

Unclean shared facilities may be due to a number of factors, including inadequate management practices of users. This inadequacy may lead to a scenario where users benefit from using a shared sanitation facility, but put little or no effort into its management. This scenario is similar to “the tragedy of the commons”, depicted by Hardin ([11]:1244), where no one wants to make personal sacrifices for the good of all users. A common good or resource is one that can be utilised by all, but that is not owned by any one user. Every user, therefore, maximises benefits from the good/resource, but the costs are shared by all [12]. For such goods, it is difficult to exclude any of the users, yet overexploitation takes away the ability of other users to use the same resource (subtractability) and eventually leads to depletion [13–15]. Applying this theory to sanitation, it may be difficult to exclude users who benefit from shared facilities, but, overexploitation, such as misuse and lack of cleaning, reduces the ability of other users to use the facility.

To minimise the challenges of common goods, Elinor Ostrom recommends the common pool resource (CPR) management principles, which are elements/conditions that encourage users to work towards a common end of ensuring the sustainability of common resources. They are:

- Boundaries
- Congruence with local conditions
- Appropriation and provision
- Collective choice arrangements
- Monitoring
- Graduated sanctions
- Conflict resolution mechanisms
- Recognition by external government authorities
- The organisation of these activities in multiple layers of nested enterprises [16–18].

These principles may not be applicable in all contexts but they work well in self-governing institutions that require coordination and collective action from users [19]. It is thus important to understand the local context within each system [20–23]. In the context of shared sanitation in informal settlements:

- A shared sanitation facility can be equated to a scarce resource.
- Management of the facility is done by the users (appropriators).
- Quality and continued use of the shared facility depends on the users’ management practices.

In urban areas, a household’s benefits from sanitation depend largely on the actions of others [24, 25] and the CPR management principles are a possible avenue to an in-depth understanding of group actions influencing shared sanitation quality. The aim of this study was thus twofold: To examine the quality of shared sanitation facilities in informal settlements, and to use the CPR principles to investigate the determinants of shared sanitation quality.

### Study area – Kisumu city

Kisumu is the main city in the western region of Kenya, with a population of approximately 420 000 people [26]. Approximately sixty percent of the city’s population lives in informal settlements [27, 28]. These settlements are faced with challenges such as lack of sanitation facilities [27]. Most residents in the settlements are tenants who commonly live in compounds. A compound is a group of several tenant households, living in individual housing units which are all under one landlord. More often than not, these housing units are constructed next to each other and they share a common yard. Compound households also share amenities such as water and sanitation facilities [29].

Statistics on access to sanitation in the settlements is scanty. An earlier study by Okurut and Charles [30] revealed that 65% of the population in the settlements have access to ‘improved’ sanitation (as defined by the JMP). Nonetheless, the study pointed out that most of these facilities did not count as providing sustainable access to basic sanitation judging from indicators such as safety, privacy, dignity, and cleanliness. Common sanitation facilities in the settlements are traditional pit latrines with a few septic tanks [31]. When these sanitation facilities are available, they are often shared within a compound [29, 32], and as mentioned, are inadequate in cleanliness, privacy, and safety [32]. Half of the compounds in the settlements lack sanitation facilities, and cases of flying toilets (the practice of defecating in a plastic bag and flinging it away) have been reported [29]. The lack of sanitation is worsened by geographical conditions in the settlements, as high water tables, loose soils and flash flooding during the rainy season lead to the collapse of pit latrines [27, 33]. The practice of flying toilets/open defecation indicates a lack of sanitation facilities, although it also may be an indication of the dysfunctional and inadequate sanitation facilities which drive residents to open defecation.

## Methods

This study adopted a case study approach. A case study aims at a comprehensive exploration/understanding of the case(s) and its interaction within specific real-world contexts ([34]:126, [35]:16, [36]:75-76, [37]:66-68, [38]), thus providing answers to the how and why questions ([39]:289). A case in this study was a shared sanitation facility, and since a number of sanitation facilities (cases) were to be studied, the study qualified as a multiple case study design ([40]:139, [41]:311). The study was limited to sanitation facilities that were shared by at least two households, within Kisumu’s informal settlements (the context).

With a case study design, more than one method of data collection is recommended in order to provide a comprehensive exploration ([35]:17), and for this reason, this study used both quantitative and qualitative methods. The study adopted an explanatory sequential mixed methods design which often begins with a quantitative phase, some initial data analysis, and then a qualitative phase in the same study area. The purpose of the qualitative phase is to further explain the results of the quantitative phase ([42]:38, [43]:224, [44–46], [47]:552).

### Quantitative stage

An initial cross-sectional study was conducted during the dry season between January and March 2014. In order to calculate the sample size, the alpha level was set at 95% and the power at 90% to increase the representativeness of the sample to the population. Based on preliminary findings [32], the difference between compounds with sanitation facilities and those without sanitation facilities was 27.8. Similarly, the standard deviation, between those with and without sanitation facilities, was 0.48. The sample size was thus calculated as 2[1.96 + 1.28]^2^0.48^2^/0.27^2^ = 67 compounds. The sample size was adjusted for a non-response rate of 20%, thus increasing the sample size to 80 compounds.

The sample was selected from Bandani, Nyalenda A, Nyalenda B and Obunga settlements. The settlements are divided into clusters, commonly called units, which are geographical sub-sections of the settlements. Nyalenda A, for instance, has Central, Kanyakwar, Western and Dago clusters [48]. Two clusters were selected from each settlement.

Since the number of compounds in each cluster was not known, transect walks with community leaders were taken in each cluster in order to estimate the number of compounds. This estimate was then divided by the required sample size from each cluster to determine the sampling interval, which in most cases was three compounds. Compounds were selected if they had a sanitation facility that was shared by households within the compound. Selection of such compounds began from one end of each cluster towards the other end. Data was collected by research assistants who worked in a group of two. In each compound, a household was selected randomly. After identifying the household, assistants established if the adult household head or their spouse was available. The purpose and requirements of the research were then explained to the respondent. If they were willing to participate, they gave their oral consent, after which the interview began.

The data collection tool used was a structured interview guide ([37]:212, [49]:344) which had closed-ended questions which the interviewer posed to the respondent. The interviewer completed the tool based on the responses given by the respondent. Respondents were asked questions relating to (among others) the type of residence, the location of the toilet and users of the toilets. After the interview, the shared sanitation facility that was used by members of the compound was inspected using an inspection tool that captured details of construction materials, location of the toilet, a rating of the cleanliness of the facility (from very dirty to very clean), and various components that define the quality of sanitation facilities as highlighted by various studies. These components of quality were hygiene factors, privacy factors and slab factors (Table 1).

**Table 1:** Quality of shared sanitation facility score sheet

Quality Factors	Yes	No
1. Hygiene		
Faecal matter on the slab?	68	57
Flies in the facility?	47	78
Smell from the facility?	97	28
A nearby hand-washing facility?	0	125
Total hygiene score (max 4)		
2. Privacy		
Does the facility have a door?	122	3
Does the door hold in place?	120	5
Does it have a locking latch?	106	19
Does it offer privacy? i.e. no cracks	110	15
Does the facility have a complete superstructure?	108	17
Does the superstructure offer privacy? i.e. no cracks on the superstructure	96	29
Does the facility have a roof?	94	31
Does the roof offer privacy i.e. no cracks?	81	44
Total privacy score (max 8)		
3. The slab and other visible factors		
Are there cracks/visible spaces on the slab?	39	86
Is the drop hole too big? (bigger than the size of a foot)	34	91
Is the drop hole open? (no evidence of a cover)	124	1
Are there standing fluids on the slab?	66	59
Is the facility full?	28	97
Is the facility semi-full?	75	35
Total slab score (max 6)		
Total quality score (max 18)		

NB: The numbers represent totals of the inspected facilities that exhibited the attributes. *n* = 125

For quality assurance and to ensure the validity of data, before beginning the survey the research assistants were trained on objectives of the research, administration of tools, handling respondents, and ethics of data collection. They were also taken through each question in the data collection tools to ensure that they understood not only the meaning of the question but also how to present the questions to the respondents. Such training ensured that all the questions were asked in a standard format. After the training, the tools were pre-tested and any issues that were not clear were rectified. Since the assistants worked in a group of two, one assistant interviewed the respondent and after the interview, the other assistant inspected the shared sanitation facilities. Both assistants and the main researcher (who was also involved in data collection) reviewed the inspection to ensure that they agreed on all aspects of the shared facility.

At the end of the survey, data had been collected from 85 compounds, with all respondents who had been selected consenting to participate. These data were transferred to Stata (v 13) for initial analysis. Some aspects of maintenance of shared sanitation had been raised during the quantitative data collection stage, showing the need to further investigate determinants of shared sanitation quality. Some of these aspects included reasons why some of the shared sanitation facilities were dirty, and how the clean facilities were kept clean. Since such aspects were beyond the scope of the quantitative survey and the data collection tool, a qualitative study was then designed using the CPR perspective.

### Qualitative stage

Driven by the inadequacies of the previous quantitative stage such as little details in answering the ‘why’ questions, this stage was carried out in December 2014. The design was informed by the characteristics of an explanatory sequential mixed methods design, in which a qualitative phase follows a quantitative phase to further explain the results obtained during the quantitative stage. Data were thus collected from the same settlements and clusters that had been selected during the cross-sectional study, but from different compounds. Just like the quantitative stage, the research assistants worked in a group of two. Compounds and household respondents were selected in a similar fashion as the quantitative stage, and respondents gave their consent for participating in the study. After combing through the cluster, more compounds were selected from neighbouring clusters in order to get more depth, variation and achieve saturation.

In each household, a guided and audio-recorded face-to-face interview was conducted with the adult household head within the compound, after which the shared facilities were inspected using the same inspection tool that had been used in the quantitative stage. One research assistant interviewed the respondent, while the other assistant (and the main researcher) recorded the interview, observed the respondent for any non-verbal communication, made notes, asked for clarification (if needed) and afterwards inspected the shared sanitation facilities within the compound.

The data collection tool was a semi-structured interview guide that had open-ended questions. Such a tool, unlike the structured interview guide that was used during the quantitative stage, allows for probing and clarification of answers, allows the researcher to guide the respondent so that they do not deviate from the main topic, and consequently, can reveal other relevant aspects that might have been missed in the tool ([36]:87-88). In addition to questions related to the residence type and number of households as in the quantitative stage, the tool had questions on the management of shared sanitation facilities. These management questions were designed using the CPR principles that had been revised to make them applicable to the local context and to sanitation, hence:

- Boundary definition of users and of the shared sanitation facility.
- Presence (or absence) of management rules/structures.
- Contribution by individuals to the common good of the shared facility (e.g. cleaning).
- Collective decision making.
- Monitoring of sanitation facilities.
- Sanctions.
- Conflict and its resolution.

The interview guide had approximately twenty-three open-ended questions that covered each of these management themes. Interviews lasted at most an hour, depending on the answers given by the respondents. Selection and interviewing continued until the point of ‘saturation’ when new information was not forthcoming. Saturation in this study was defined by the principle that the sample size ought to be ‘large enough’ to provide a thick description and support convincing conclusions, but not too large to hinder a thorough analysis ([37]:421,425, [39]:162). Selection therefore continued until a total of 40 respondents had been interviewed and the 40 toilets within their compounds also inspected.

To ensure the quality of the data collected, the same research assistants were involved in this second stage of qualitative data. They were again trained on the new data collection tool and its administration. The tools were pre-tested to ensure that the researchers and respondents understood the questions and that the questions were asked in the same format. A pilot study was initially carried out to assess the applicability of the common pool resource management principles to shared sanitation and to design the interview questions. Data collection teams always had a male and female to cater for circumstances when a respondent needed to be interviewed by someone of the same gender.

Overall, this study was strengthened by the use of quantitative and qualitative methods of data collection. Whereas the quantitative data collection methods identified the problem (quality of shared sanitation facilities), the qualitative methods provided an opportunity for a finer explanation of the issues identified during the quantitative stage (reasons explaining the quality of shared sanitation facilities). Having an initial quantitative stage and a follow up qualitative stage in the same settlements increased the sample size, decreased bias and increased the representativeness of the sample to the population.

#### Data management and analysis

Quantitative data from all the inspected sanitation facilities were entered in EpiInfo and checked for any errors before transferring to Stata (v 13) for analysis.

Just like previous studies that have calculated the quality of sanitation as a score of the various attributes [5, 7], the quality of shared sanitation facilities was calculated as a score, summed from each of the three main factors (hygiene, privacy, and state of the slab). For hygiene and slab factors, if the answer to any of the questions was no, the facility scored 1, otherwise, it scored 0. However, it was the reverse for the availability of a hand-washing facility: 1 if yes, and 0 if no. For privacy-related factors, the score was 1 if the answer to any of the questions was yes, and 0 if otherwise.

To examine the determinants of quality, a standard multiple linear regression was performed with the total quality score as the dependent variable. The independent variables were settlements, the location of the toilet, superstructure and slab materials, toilet users, and number of households sharing a toilet. Two hypotheses were being tested: that poor-quality construction materials of the superstructure and the slab lead to lower quality of shared sanitation facilities; and that more households sharing a sanitation facility result in lower quality of shared facilities.

For the qualitative phase, initial analysis of data began while conducting field work to identify and refine any emergent issues that may have been missed and needed follow up in subsequent interviews. After data collection, all recordings were replayed by the main researcher in order to get an overall understanding of each respondent’s story. The interviews were transcribed verbatim in Microsoft Word, and the main researcher then re-read the transcripts. The transcripts were then transferred to ATLAS.ti software. In ATLAS.ti, analysis followed a thematic content analysis approach [50]. The transcripts were first coded based on frequently appearing words or issues (for instance, locking latrines). The codes were then merged into families which were the CPR themes that had been identified a priori (such as defined boundaries of a compound). The themes were then summarised in a matrix, (referred to as the Primary Documents table in ATLAS.ti, and presented as Table 2) which presented the frequencies of these themes and codes within the shared sanitation facilities. Such a matrix revealed some cases that were ‘out of the norm’, commonly referred to as deviant cases. Such cases often prompted the researcher to revisit the transcripts, compare the coding, and relate the cases to the theories in order to obtain a deeper understanding. This process led to finer explanation on possible reasons for the quality of shared sanitation facilities experienced during the quantitative stage. The convergence of the quantitative and qualitative findings was then reconciled at the point of interpretation of the data (analytic or interpretative integration) [51] by linking the CPR theory to shared sanitation quality in order to provide a richer discussion.

**Table 2:** Descriptive summary of findings

Variables	Frequency (%)
Area	
Bandani	29 (23.2)
Nyalenda A	31 (24.8)
Nyalenda B	34 (27.2)
Obunga	31 (24.8)
Roof material	
None	31 (24.8)
Iron sheet	94 (75.2)
Superstructure material	
Iron sheet/mud/wood	61 (48.8)
Bricks/stone	64 (51.2)
Floor/slab material	
Mud/wood	15 (12)
Stone /slab	110 (88)
Location of toilet	
Outside compound	25 (20)
Inside compound	100 (80)
Toilet users	
Owner and tenants	38 (30.4)
Tenants and caretaker	38 (30.4)
Tenants only	49 (39.2)
Rated cleanliness	
Very clean	15 (12)
Clean	30 (24)
Dirty	53 (42.4)
Very dirty	27 (21.6)
Number of households sharing	Mean 8.4 (2-27) Std dev 4.7
Hygiene score	Mean 1.3 (0-3) Std dev 1.1
Privacy score	Mean 6.7 (2-8) Std dev 1.6
Slab score	Mean 2.9 (0-6) Std dev 1.4
Total quality score	Mean 10.9 (5-17) Std dev 3.1

## Results

### Quantitative results

Tables 1 and 2 present a summary of the study findings. Apart from describing the aspects defining quality in the inspection tool, Table 1 also presents the total count of facilities that exhibited these quality aspects.

All inspected sanitation facilities were pit latrines shared by averagely eight households (Table 2). Most of these facilities (64%) were dirty (either slightly dirty or very dirty). Compounds with clean toilets had an average of seven households sharing a toilet, while dirty sanitation facilities had a mean of nine households.

Regression analysis results summarised in Table 3 indicated an inverse relationship between quality and number of household users (*p* = 0.04; CI-0.22- -0.001). Sanitation facilities constructed with a brick superstructure had two scores of better quality compared to sanitation facilities with iron sheets/mud/wood superstructure (*p* < 0.01; CI 0.91-3.11). Thus the hypotheses that shared sanitation facilities with more people have lower quality, and that poor construction materials lead to lower quality of shared sanitation facilities were accepted. However, for the slab material, there was no evidence to reject the null hypothesis (*p* = 0.13, CI-0.39-2.99), leading to the conclusion that there was no difference in quality between the slab construction materials. In addition, as shown in Table 3, regression results indicated no quality difference based on type of residence (tenants only or tenants with caretaker). The R-squared value of the final regression model suggested that the variables in the model explained only 26% of the shared sanitation quality. It was assumed that more factors, i.e. management practices, would further explain the quality of shared sanitation facilities.

**Table 3:** Summary of regression results of determinants of quality of shared sanitation facilities

Variables	Regression Coefficient	Std Error	*P* values (CI)
Number of households sharing the facility	-0.11	0.05	0.04 (-0.22 - -0.001)*
Toilet located within the compound	0.9	0.67	0.19(-0.45 -2.19)
*Superstructure* Bricks/stone superstructure	2.01	0.56	<0.01 (0.91-3.11)*
*Slab material* Concrete/stone slab	1.30	0.86	0.13 (-0.39-2.99)
Residence type/users			
Tenants and caretaker	-0.15	0.67	0.81 (-1.48–1.18)
Tenants only compounds	-0.85	0.64	0.19 (-2.13-0.42)
Area			
Nyalenda A	-0.80	0.77	0.31 (-2.33-0.72)
Nyalenda B	-0.16	0.75	0.83 (-1.64–1.33)
Obunga	-0.13	0.80	0.87 (-1.71–1.45)
R^2^	0.26		
F(9, 115)	4.4		
Prob (F-statistic)	0.00		
*N*	125		

*Significant at *p* = < 0.05

### Qualitative results/applicability of CPR principles

Majority of the respondents were female tenants, with a few male tenants and female landladies. Most of these respondents were middle aged, and had children or lived in compounds where their neighbours had children. The CPR principles were exhibited in various ways:

#### Boundary definition

Boundaries were demarcated in various ways: Toilets were situated within fenced and/or gated compounds and they were locked with padlocks. In compounds where toilets were locked, each household had a copy of the keys or one key was shared by at least two households. In other cases, keys were situated at strategic positions (e.g. on a wall) where they were accessible to households within the compound. Users acknowledged that toilets were locked to keep intruders, who often left the toilets dirty, away.

*“We keep them [toilets] locked because other passers-by and people from neighbouring compounds would want to use them.”* [A Female tenant]

Cases of users losing their keys, which eventually led to the toilets not being locked, were also reported. Such toilets were an easy target for illegitimate users, especially if they were not within fenced or gated compounds. The breaking of padlocks (in order to use the toilets) and stealing of materials used for the construction of the facilities, were also reported.

Often times, dirty facilities were left open for all to use, including members of other compounds. Users from compounds with such dirty and ‘open for all’ toilets did not feel the need to block outsiders from using their facilities.

*“How and why should one prevent outsiders from using such a toilet? It is already too dirty.”* [A male tenant]

#### Cleaning arrangements and rules of use

There were varied cleaning structures or patterns. Defined cleaning structures were commonly in the form of a duty rota, and each household had a specific day(s) when they cleaned toilets. It was not a written rota per se, but rather households followed an order (e.g. arrangement of houses within the compound) that ensured that all users participated in cleaning the toilet. Such structures worked best in compounds with fewer households who had good relations among themselves. Women were mostly responsible for cleaning the toilets.

*“I clean on Monday and Wednesday, and the others also have two days of the week when they clean.”* [A female tenant]

In some compounds with live-in landlords, the landlords cleaned the toilets without involving tenants.

In other compounds, even with defined cleaning arrangements and rules of use, some users did not perform their cleaning duties as expected. At other times, when the person responsible had cleaned the toilet, other compound members soiled them, which led to other users not carrying out their cleaning responsibilities. A female tenant explained that it was common for other users to soil the toilets after the person responsible had cleaned them. When asked what she did in such a situation, she said:

*“If someone else soils it [the toilet], I will ask them to clean it; else I just leave it dirty.”*

In compounds where cleaning rules and/or management structures were not well defined, toilets were often left dirty and would only be cleaned by any member who volunteered to clean.

*“Nobody cares about this toilet … whoever is willing to clean it will do it …”*

Such toilets remained dirty, often for a number of days, before someone volunteered to clean. When sanitation facilities were left dirty, most respondents mentioned using their neighbour’s facilities. A few admitted to using flying toilets or defecating in the open.

Often times, the common cleaning practice was to simply pour dirty soapy water, which had been used to clean clothes, over the toilet slab. Women often volunteered and took on the responsibility of cleaning sanitation facilities in order to protect their children from using unhygienic facilities. One female tenant explained why she cleaned the toilet in their compound every day without relying on anyone:

*“I clean the toilet … because I have children … I do not want them to use a dirty toilet.”*

In most compounds, it was commonly felt that cleaning toilets was a woman’s responsibility, and thus men were sometimes exempted from cleaning/management activities. However, in other compounds, all users (both male and female) were required to clean the toilets

It was also noted that in some compounds, there had previously been a cleaning and/or management structure that was abandoned when users did not adhere to the rules. When rules were not adhered to, the toilet was either left dirty or would be cleaned by anyone who volunteered.

*“We previously had a rota…. but members started complaining…they refused to clean the toilet. ….Eventually, no one cleans it.”* [A female tenant]

#### Collective decision-making

Meetings were held in some compounds, and all members were required to attend. During such meetings, sanitation issues affecting members were raised and discussed. Such meetings often led to collective decisions and the formulation of rules for the management of sanitation facilities.

*“We, the tenants, held a meeting and all agreed to it [toilet cleaning plan]* [a female tenant]

These meetings were easier to coordinate in compounds with fewer households, or in compounds with a leader, such as a landlord or the caretaker (A person appointed by the landlord to be in charge of all tenants in a compound). The leader ensured that all users participated in decisions and that they carried out their duties as agreed upon in the meeting. On the other hand, it was difficult to arrive at a consensus in compounds with many members, even when there was a leader, often due to differences in opinion, uncooperative members, or the unavailability of all members during decision-making meetings.

#### Monitoring

Monitoring was done to check on illegitimate users as well as on the condition of the toilets. In tenant-only compounds, one of the tenants would sometimes act as the leader, and in compounds with a live-in landlord, the landlord took up the responsibility. Other compounds had caretakers who took up this role. Monitoring was done in various ways. For example, when residents sat outside their houses during the day, they would easily identify any illegitimate users. In some tenant-only compounds, tenants themselves acted as monitors, a practice which was successful when they had good relations among themselves. A female tenant, who lived in a compound of three households, mentioned that they did not have any one person responsible for monitoring their toilet, but that they all did it together. When asked how they do it, she explained:

*“We are all responsible, for example, if someone from a neighbouring compound comes to me asking to use the toilet, my next-door neighbour will not allow them.”*

Compounds with defined boundaries such as fences and gates needed less monitoring, as it was not easy for outsiders to sneak in and use the toilets; unlike compounds without boundaries.

#### Conflict and its resolution

Cases of conflict were reported in instances when users soiled sanitation facilities after they had been cleaned. Conflicts were common among women, especially if children dirtied the toilets and their guardians did not clean them. These conflicts were at times physical fights, disagreements, exchange of words, or quarrels among compound members. At times conflict was experienced in a subtle way, for example,

*“People sulk at each other, others talk ill of those who dirty the toilet.”* [A female tenant]

Conflicts were resolved in various ways, including discussions with the concerned parties individually or collectively; or involving a third party – often the leader. It was also noted that without cleaning or management rules, users in compounds with dirty toilets often experienced little or no conflict since no one was in charge of cleaning the toilets.

#### Sanctions

In most compounds, sanctions were administered by other compound members or the leader. Reported sanctions included buying new padlocks when keys were lost, or being forbidden to use the toilet if anyone lost the keys. Sometimes a landlord gave un-cooperating tenants a warning when they refused to abide by cleaning rules. If such tenants continued being uncooperative, they were asked to vacate the compound. A male landlord explained that he was very vigilant in ensuring that the tenants in his compound kept the toilet clean. When asked what he did if there were any tenants who did not follow the set cleaning rules, he explained:

*“I give the [uncooperative] tenant three warnings, after which I ask them to vacate the compound.”* [A male Landlord]

In extreme cases, one landlord explained that when tenants stubbornly refused to abide by the toilet rules in the compound such as not cleaning the toilet after they soil it, they were reported to the local chiefs.

Table 4 summarizes the application of the CPR principles in the clean and dirty sanitation facilities.

**Table 4:** Applicability of CPR principles in shared sanitation facilities in Kisumu’s informal settlements^a^

	Management principles	Clean	Dirty
1	Defined boundaries of compound and toilet	17	7
2	Defined cleaning arrangements	15	7
3	Users participate collectively in decisions	9	4
4	Users experience conflict	1	8
5	Conflict resolution mechanisms	1	2
6	Monitoring of the toilet and users	12	9
7	Defined rules of use	5	2
8	Sanctions	6	2
9	Total number of inspected sanitation facilities	17	23

^a^Summarised from Primary Documents table of ATLAS.ti software

## Discussion

Defining the quality of shared sanitation facilities is critical if they are to be considered as improved sanitation by the JMP. In this study, shared sanitation quality was measured as a total entity that included the roof, superstructure, as well as hygienic conditions. Most of these indicators were used by other studies [5–10, 52] that aimed at assessing the hygienic conditions of sanitation facilities. The advantage of using all these indicators as used in this study is that the measure of quality is all-inclusive, and not only focused on hygienic aspects. For example, anyone using a shared sanitation facility would be more comfortable to use one that is not only clean but also offers privacy and shelter from weather conditions such as rainfall.

Results of this study reveal that household shared sanitation facilities were pit latrines, some of which had iron sheet as the superstructure material. Superstructures made out of iron sheets were likely to have a wooden slab and were often not hygienically clean compared to facilities with superstructure made from bricks which had better sanitation quality. Unlike iron sheets, bricks are more durable, they are likely to have fewer crevices, and thus offer better privacy. Moreover, due to a high water table that leads to the collapse of pit latrines in the study area, it is unlikely that one would construct a sanitation facility with a superstructure made of bricks and use poor quality slab material because the toilet easily collapses. Similar findings were reported in Uganda, where latrines with plastered brick superstructures were structurally sound and showed little signs of collapse during the rainy season [9].

Quantitative results further showed that the facilities performed better in privacy aspects compared to hygiene and slab aspects. A closer look at the scores of hygiene and slab aspects (such as faecal matter and fluids on the slab which can be a public health risk) points to the fact that the facilities were not adequately maintained. Similar results are reported in Western Kenya [5] and Tanzania [7] where although most facilities had complete superstructures, they were not hygienically clean as evidenced in their hygiene and slab aspects. Better performance of privacy aspects reveals that more attention is usually given to the provision of the ‘structure’ (hardware), and the poor performance in hygienic conditions points to less attention being given to the behaviour of users. Sustainability in sanitation is not only about the provision of hardware aspects. A great extent of shared sanitation quality is explained by ‘soft’ factors that are behavioural [53] and related to maintenance/management, and thus attention also needs to be directed to users’ behaviour and practices.

To further examine the role of sanitation practices, findings revealed that the quality of shared sanitation decreased with an increase in the number of users, similar to findings from informal settlements in Uganda [53–56]. The relationship between increasing number of users and decreasing quality, especially in informal settlements, is explained by users’ behaviour. Studies have related the cleanliness of shared sanitation to individual behaviour [10, 52, 55, 57] such as having the intention to clean sanitation facilities. However, qualitative findings from this study suggest that all users may not share the same intent or have the same attitude. In addition, one individual’s effort may not be as productive as a group’s effort, and it is, therefore, important to investigate group dynamics and behaviour, especially with shared sanitation. Group dynamics, provide a more holistic picture as opposed to an individual’s actions, which may or may not have as significant an effect on quality as the actions of a group would, hence the application of the CPR principles.

One of the CPR principles is boundary definition, and in the context of shared sanitation in informal settlements, it included strategies like locking shared toilets and having the toilets located in fenced compounds to keep intruders away. Such practices have also been reported in informal settlements in Nairobi [58], Uganda [52, 53] and India [59]. Defining boundaries is crucial because it identifies legitimate users, and it becomes easier to coordinate efforts among the legitimate users. Having a defined user group is also important in defining and implementing management structures and practices like cleaning, collective decision making, and monitoring. A defined user group and management structure do not, however, guarantee that facilities will be in proper hygienic conditions. Results of the current study, as well as studies from Nairobi [58] and Bangladesh [60], show that some management system such as cleaning rotas break down after some time, implying that there is more that explains the quality of shared sanitation other than the users, defined boundaries and defined management structures.

In addition to users, defined boundaries, and defined management structures; cooperation from and among users is equally vital. Cooperation results when users are in communication, and it ensures that sanitation facilities are kept clean, the defined structures are implemented, monitoring and sanctioning are implemented, and conflicts are resolved. One way of attaining cooperation in groups is through collective decision making. Collective decision making ensures that decisions that are arrived at are favourable to everyone. Qualitative results, for example, indicated that residents who lived in compounds with dirty facilities rarely made decisions collectively. When users are in agreement, it is possible and easier to organise for collective action to ensure that shared facilities are of acceptable quality. McGranahan [24] also highlights the importance of collective action in sanitation by noting that when there is collective action, it becomes possible to solve local sanitation challenges.

It is also indicative from this study that management practices are interrelated, each working with another to influence the quality of sanitation facilities. For example, with a defined user group, proper management structures, and collective decision making; monitoring, implementing sanctions, and conflict resolution are easier. These practices were rarely carried out in compounds with dirty sanitation facilities in this study. Therefore, it is possible to effect some of the CPR management practices but still have dirty sanitation facilities.

Such a scenario where some management principles are implemented but shared facilities are not hygienically clean can partly be related to the number of users. Relating back to the issue of numbers, this and other studies [53–56] show decreasing sanitation quality with increasing number of users. A large number of users is difficult to coordinate, make decisions collectively, and implement rules. It is difficult for people in large groups to trust all other participants, hence making it easier to free ride on the actions of others [18]. Consequently, sanctions may not be easily implemented and effected, and conflict, which is mostly due to the neglect of responsibilities may arise, as reported in India [60] and Ghana [61]. On the other hand, it may be possible to have a smaller number of users and also have dirty sanitation facilities. Such a situation may arise when for example, the few users do not have defined boundaries and management structures and do not collectively make decisions.

On the issue of numbers and in line with the discussion on classifying shared sanitation as improved or not, some authors have proposed minimum or maximum numbers allowable for sharing. Gunther et al.’s [56] study recommended that facilities be shared by a maximum of four people. Kabange and Nkansah [3] suggest sharing with 2-3 families. One limitation of this study, though, is that it was not possible to establish a threshold number. Although it is clear that shared sanitation quality decreases with increasing number of users, it should be noted that the size of a household is crucial in defining a threshold. For example, compounds may have a number of housing units, which may be occupied by one individual in each unit. Individuals in such a compound may be able to work collectively and keep their sanitation facilities clean. Alternatively, a compound with a similar number of housing units that are occupied by families with an average of five members may have dirty shared facilities. Again, as earlier discussed, it is also possible to have fewer households sharing sanitation facilities but still have unhygienic and dirty facilities. Therefore, focusing on numbers alone is not enough to make recommendations; other determinants beyond the numbers are also important.

Aside from the already discussed CPR principles, another determinant of shared sanitation quality is the importance of good relations among users of sanitation facilities. With good relations, users can, for instance, take on other users’ roles in cleaning sanitation facilities, or they are likely to resolve issues amicably thus resulting in less conflict. Good relations between landlords and tenants may also lead to productive management practices, even without the use of sanctions or monitors. For example, some individual tenants participated in the management of shared sanitation because it was a norm that every user should take part in management. Non-participation in behaviour such as cleaning can be viewed as ‘abnormal’ and is part of the reason why landlords were ready to evict non-cooperative tenants. Hence, it is possible that even when sanitation facilities are shared by many households and it may be expected that their sanitation facilities be dirty, there may be social rules and norms that guide users in ensuring that these facilities are kept clean. The CPR literature also proposes that where there are no rules, social ties may reduce conflict and facilitate the development of rules or social norms, which lead to the growth of beneficial behaviour that encourages cooperation [62–64].

Further noted in this study were the aspects of gender and the role played by individual efforts that contributed to the good quality of shared sanitation. Results showed that women were more likely to clean sanitation facilities, rather than men. In addition, they volunteered to clean sanitation facilities because they had young children who would be exposed to the risk of disease. Women have often been responsible for sanitation, including cleaning, also reported among users of shared facilities in Uganda [65] and India [60]. However, when users depend on actions of specific individuals and do not put in their own effort, it is possible that such beneficial actions of specific individuals may stop when circumstances change e.g. when children grow up, or when users relocate to other areas. Management of the shared sanitation facility may then end up being no one’s responsibility. If only specific individuals participate in a group’s common good, the practice may not be sustained long enough to ensure continued use of shared sanitation facilities. Eventually, as noted by Tumwebaze [54] poor-quality sanitation facilities may not be used and users may resort to practices such as open defecation.

## Conclusion

This study has highlighted that the quality of shared sanitation facilities is not only influenced by hardware aspects but also software aspects. Hardware aspects include construction materials, while software aspects include the behaviour and practices of users. Software factors, which were investigated using common-pool resource management principles, show the importance of group dynamics and practices because they determine the quality of shared sanitation facilities. Shared sanitation facilities should be located where illegitimate users will not have access, and the legitimate users should have a management system that they agree upon collectively. With cooperation, collective action is possible, as the group works together to ensure that sanitation facilities are in good condition. Such an environment also enables the development of social norms that guide other users towards responsible behaviour with regard to shared sanitation facilities. The CPR principles provided useful insights into the complex dynamics of shared sanitation management. Emergent from this study is that, in relation to sanitation in informal settlements, focusing only on numbers may suggest fewer number of users per facility, and consequently more sanitation facilities in informal settlements, both of which may not be feasible. Attention should also be directed at practices that ensure cooperation among users for their common good. Otherwise, ‘access’ to sanitation does not always mean ‘use’ of sanitation facilities.

### Policy implications and recommendations for further studies

Policy makers and stakeholders, such as public health departments, should ensure that efforts are not only directed at increasing access but also at ensuring that shared facilities are in useable hygienic conditions. These efforts should involve stakeholders, such as landlords, tenants, and local leaders. As this study suggests, shared sanitation facilities can be kept hygienically clean if there is collective effort from users. Therefore, for policy development, the focus should not only be on number of users sharing a facility but also on the behaviour and practices of the users. With such consideration, it means that if managed adequately, household shared sanitation in Kisumu’s informal settlements may be considered as improved sanitation. Development efforts should in addition to the provision of sanitation, also include aspects of safe and hygienic use of shared sanitation facilities and proper disposal of human excreta. Hygienic use of sanitation facilities will ensure that there is sustained use of shared sanitation facilities. Follow-up studies could be carried out to determine the number of users who can share sanitation facilities while ensuring that there is cooperation and coordination amongst them towards a common goal.

### Limitations

The study was carried out during the dry season, and it is possible that the results may be different during the rainy season. Being a case study design, the findings of this study are applicable within the context of Kisumu’s informal settlements. This study may then be a basis for comparison with studies (perhaps with larger sample size) from other informal settlements.

## Abbreviations

CPR: Common pool resources
JMP: Joint Monitoring Program
NACOSTI: National Commission for Science and Technology
REC: Research Ethics Committee
SDG: Sustainable Development Goal
UNICEF: United Nations Children’s Fund
VIP: Ventilated Improved Pit latrine
WHO: World Health Organisation
